# Predictors of Teenage Fatherhood Among Justice-Involved Adolescents

**DOI:** 10.3390/ijerph22121801

**Published:** 2025-11-28

**Authors:** Naomi McGoldrick, Colleen Sbeglia, Lauren Wyckoff, Paul J. Frick, Laurence Steinberg, Elizabeth Cauffman

**Affiliations:** 1 Department of Psychology, University of California Riverside, Riverside, CA 92521, USA; 2 Department of Psychology, University of California Irvine, Irvine, CA 92697, USA; colleeb@uci.edu (C.S.); pepper1997@cox.net (L.W.); cauffman@uci.edu (E.C.); 3 Department of Psychology, Louisiana State University, Baton Rouge, LA 70803, USA; pfrick@lsu.edu; 4 Psychology and Neuroscience, Temple University, Philadelphia, PA 19122, USA; lds@temple.edu

**Keywords:** teen father, justice-involved youth, teen pregnancy

## Abstract

Justice-involved boys are more likely to become teenage fathers than their community peers. This is linked to numerous negative outcomes, including increased delinquent behavior. To help legal practitioners better identify which boys are at risk of becoming a teen parent, this study identifies factors at the time of boys’ very first arrest that prospectively predict their odds of becoming a teen father. Data were drawn from a longitudinal study of 1216 adolescent boys at the time of their first arrest. Binary logistic regression models were used to predict the odds of becoming a teen father across three domains: individual factors, social and contextual factors, and risky behaviors. Approximately 15% of the total sample (*n* = 171) reported becoming a teen father after their first arrest. At the time of first arrest, poorer neighborhood conditions, increased peer delinquency, substance use, and self-reported offending history increased the odds that boys would become a teen father. Additional models indicated that substance use was the strongest driver of teen fatherhood. However, all factors failed to reached significance once condom use was included in this model. Practical implications for policymakers are discussed, along with suggestions for interventions to reduce teen pregnancy.

## 1. Introduction

In 2023, the United States hit a historical low in teen birth rates with 13.2 births per 1000 females [1]. Despite this decline, the United States still has one of the highest teen birth rates among industrialized countries, with data suggesting that at least 70 percent of pregnancies among youth aged 15–19 are unplanned [2]. Teen pregnancies may be especially pronounced among youth who are justice-involved, with studies indicating that their teen pregnancy rate exceeds five times the national average [3]. Identifying precursors to teen pregnancy among justice-involved youth is an important public health issue, as parenthood among teens is linked to various adverse developmental and psychosocial outcomes for both young parents and their children [4]. Prior literature has examined risk factors for teen parenthood, but the majority of studies have focused on girls who become teen parents [5], despite the fact that males can cause multiple pregnancies within a single pregnancy time window. Among the relatively few studies that have examined teen fatherhood, none to our knowledge have specifically identified factors which precede teen fatherhood in a justice-involved sample. Given the small number of studies on the predictors of teen fatherhood and elevated rates among justice-involved boys, it is important to prospectively examine factors associated with becoming a justice-involved teen father.

### 1.1. Public Health Concerns Associated with Teen Fatherhood

Teen parenthood, defined as bearing a child before the age of 20, has consistently been recognized as a risk factor for not only the parents but also for their children, who face increased likelihood of low academic achievements, greater engagement in antisocial behaviors, and teen parenthood themselves [6,7]. More specifically, Alio and colleagues’ study revealed that infants born to adolescent fathers are at increased risk of being born preterm and smaller for their gestational age [8], introducing potential developmental and long-term health challenges. Moreover, compared to first time adult fathers, teen fathers reported lower sense of competence and less concern for child-rearing responsibilities and quality of the father-child relationship [9]. Overall, these findings underscore the unique challenges faced by teen parents, particularly teen fathers, and their children.

Given the wide range of poor outcomes related to teen parenthood, it is critical to also examine the diverse factors that contribute to the likelihood of becoming a teen parent. One study revealed that persistent antisocial behaviors during childhood and adolescence, as well as reports of depression during mid-adolescence were associated with increased likelihood of becoming a teen mother [10]. Moreover, both engagement in substance use and perceived peer substance use have also been linked to earlier sexual debut and teenage childbearing [11]. With the majority of existing literature investigating precursors of teen motherhood, research exploring factors leading to teen fatherhood remains limited, especially among justice involved boys.

### 1.2. Teen Fatherhood and Justice Involvement

Justice involvement itself appears to be linked to teen pregnancy in both females and males. Girls who have been involved in the juvenile justice system are more than three times as likely to become a teen parent compared to girls who have not been arrested [12] and roughly one-third of incarcerated boys are estimated to be teen fathers [13]. While prior research has yet to examine what predicts teen parenthood for justice-involved boys in a longitudinal framework, correlational work has identified factors which may put teen boys at risk. Studies examining the correlates of teen fatherhood often organize potential predictive factors into distinct domains which address multiple ecological levels, a framework informed by Bronfenbrenner’s ecological systems theory [14]. Application of this model considers not only the individual’s agency in risky sexual behaviors but also identifies important social and contextual level factors that underlie such risks [15]. In perhaps the most comprehensive study on justice-involved teen fatherhood to date, Khurana and Gavazzi examined contextual risk factors across multiple domains that were linked to teen fatherhood among juveniles who had been charged with a crime [16]. Their findings revealed that teenage fathers were exposed to a greater number of ecological risk factors (e.g., history of family incarceration, experience in child protective services) than their peers who did not have a child. However, because the risk factors were studied after the teen became a father, it is not clear what risk factors preceded teen fatherhood.

The possibility of teen pregnancy and parenthood is likely influenced by a large number of factors. Organizing these factors into distinct domains may help policymakers understand which types of interventions are most effective (e.g., should they focus on individual-level factors like mental health, or social factors like peer influence?), and which factors may be most efficient to target (e.g., within social factors, do peers or parents have a bigger influence?). For justice-involved boys, these factors can be broken down into individual factors, social and contextual factors, and risky behaviors.

Individual characteristics may play a central role in adolescents’ engagement in risky sexual behavior, particularly during this developmental stage characterized by impulsivity [17]. These associations may be especially pronounced among justice-involved boys, who may face additional unique vulnerabilities. Knowles and colleagues’ study reveal that individual factors, namely impulse control and future expectations, are linked to risky sexual behaviors among justice-involved boys [18]. More specifically, diminished future expectations and poorer impulse control were linked to more frequent casual sex and inconsistent condom use [18]. Other studies report that sensation seeking, impulsive decision-making, and self-esteem are also associated with risky sex among community teens [19,20]. Heightened self-esteem was associated with early sexual debut (before age 16) and less frequent condom use [19], while elevated sensation seeking and impulsive decision-making were linked to engagement in unsafe sexual behaviors, including alcohol and marijuana use before sex [20]. This may be explained by the low concern for potential consequences, perhaps resulting from a lack of positive expectations for the future, or a desire to obtain approval from peer groups [18,21].

In addition, social relationships may impact the likelihood of becoming a teen father. Greater numbers of teen pregnancies have been observed among youth who are exposed to deviant peers [22], and low levels of parental monitoring are linked to increased high-risk behaviors, including unprotected sex [23]. Physical contexts may also increase risk, with neighborhood disadvantage, and particularly exposure to neighborhood violence strongly predicting teen pregnancy [24]. Conversely, contextual factors may also buffer against risky sexual behaviors. School connectedness, for instance, appears to reduce risk for adolescent fatherhood, especially among youth exposed to trauma [25].

Finally, risky behaviors are associated with teen parenthood. Offending behavior often co-occurs with teen fatherhood, with evidence suggesting that higher levels of past offending are associated with higher risk [13,16]. Similarly, substance use appears to increase the likelihood of teen pregnancy, with substance use preceding and continuing throughout teen parenthood [26]. Despite the numerous factors that may specifically increase risk for justice-involved boys, it is important to remember that risky sexual behaviors (e.g., not using condoms) greatly and consistently increase the odds that youth will become teen fathers. Using data from more than ten thousand boys in the National Longitudinal Study of Adolescent Health, having sex without a condom increased the odds of teen fatherhood by more than 70%, while having stronger beliefs about the importance of using contraception decreased the odds by nearly 50% [27]. Notably, increased substance use during adolescence and young adulthood has been consistently linked to early sexual initiation, inconsistent condom use, and multiple sex partners, thereby elevating the risk of unplanned pregnancies and sexually transmitted infections [28]. Thus, it is critical for policy considerations to account for sexual risk-taking alongside factors more specific to justice involvement.

### 1.3. The Present Study

Given the heightened prevalence of teen fathers among justice-involved boys, understanding whether there are unique factors that are responsible for teen fatherhood among justice-involved boys may help public policymakers design and support interventions which prevent elevated rates of teen fatherhood. While previous studies have explored risk factors to teen fatherhood in community samples or focused on the detrimental outcomes of becoming a teen father, this study seeks to identify factors at the time of a teen’s very first arrest that are prospectively associated with him becoming a teen father. By examining factors at youths’ first point of contact with the justice system, legal policymakers may be better able to identify and support boys who are at the highest risk of teen fatherhood and focus on modifiable factors that can mitigate the risk of unintended teen pregnancies.

## 2. Materials and Methods

### 2.1. Sample

Data were drawn from the Crossroads Study, a longitudinal study of 1216 male youth who were aged 13–17 years (*M* = 15.3, *SD* = 1.3) at the time of their first arrest for a low-level crime (e.g., misdemeanor offenses such as theft or vandalism). Participants were recruited from three sites: Philadelphia, Pennsylvania (*n* = 533); Orange County, California (*n* = 532); and Jefferson Parish, Louisiana (*n* = 151). Approximately 46% of youth self-identified as Hispanic/Latino, 37% as Black/African American, 15% as White, and 2% as another race, which reflects the disproportionate number of minority youth who come into contact with the juvenile justice system. See Cauffman et al. [29] for more details.

### 2.2. Procedures

The study procedures were approved by the Institutional Review Board (IRB) at all three study sites (the Institutional Review Board of University of New Orleans, 20107867, 22 December 2010; the Institutional Review Board of Temple University, 20107867, 22 December 2010; the Institutional Review Board of University of California, Irvine, Protocol # 20107867, 11 March 2025). A Privacy Certificate issued by the Department of Justice protects participants’ identities and responses from subpoenas, court orders, or any other type of involuntary disclosure. Informed parental consent and youth assent were obtained from all participants, and youth were told that participation was voluntary. Participants were initially interviewed within 6 weeks after their first arrest (“baseline”) between July 2011 and May 2013. Follow-up interviews were then conducted every 6 months after their initial interview for the first three years, annually for two years (years 4 and 5), and then every two years after that (years 7 and 9), for a total of 10 interviews. These were conducted by a trained research assistant using a secure, computer-administered program. Participants were paid for each interview, with increased payment amounts at each timepoint to encourage study retention.

### 2.3. Measures

#### 2.3.1. Outcome Variable

Teen fatherhood. At each timepoint across the 9-year study period, youth were asked if they had any children (“Do you have any children?”). For youth who ever indicated “yes,” data were examined to identify the first interview timepoint that they reported this, and what age they were during that interview. Participants who were 19 years old or younger during the interview that they first reported being a father were coded as a teen father (Teen father = 1, Not a teen father = 0). Although youth legally become adults at the age of 18, using a cutoff of age 19 or younger is consistent with prior research [16].

#### 2.3.2. Individual Predictors

Impulse control. Impulse control was measured with an 8-item scale (WAI; [30]) that assessed an individual’s ability to control impulses within the context of external constraints (e.g., “I say the first thing that comes into my mind without thinking enough about it”). Responses were averaged from a 5-point scale from 1 (“True”) to 5 (“False”) and were reverse coded so that higher scores reflect greater impulse control. These items were measured during the baseline interview (*M* = 3.3, *SD* = 0.85, α = 0.74).

Sensation seeking. Using items that assessed thrill or novelty-seeking behaviors (e.g., “I like doing things just for the thrill of it”), sensation seeking was measured with a 6-item scale (SSS; [31]). Participant’s responses were counted and summed as “True” (1) or “False” (0) during the baseline interview (*M* = 3.8, *SD* = 1.70, α = 0.70).

Future orientation. Future orientation was measured with a 15-item scale (FOI; [32]), that assessed the degree to which youth engage in planning and consideration of the future (e.g., “I will give up my happiness now so that I can get what I want in the future”). Responses were averaged from a four-point scale from 1 (“Never True”) to 4 (“Always True”). These items were measured during the baseline interview (*M* = 2.5, *SD* = 0.50, α = 0.66).

Self-esteem. Self-esteem was measured by a 10-item scale (RSES; [33]), which asked how much youth agreed with statements on a four-point Likert Scale, ranging from 1 (“Strongly Disagree”) to 4 (“Strongly Agree”). Items assessed an individual’s feelings of self-worth and self-acceptance (e.g., “I take a positive attitude toward myself”) and responses were summed from the baseline interview (*M* = 21.3, *SD* = 4.4, α = 0.83).

#### 2.3.3. Social and Contextual Predictors

Exposure to violence. Violence exposure was assessed using a 13-item scale (ETV; [34]) that counted the number of types of violence youth had either directly experienced or witnessed. Youth responded to items measuring direct victimization (e.g., “Have you ever been beaten up?”) and witnessing violence (“Have you ever seen someone else get chased where they might be seriously hurt”) during the recall period with a “yes” (1) or “no” (0). Responses were counted and summed at baseline (*M* = 2.8, *SD* = 2.6). Approximately 77% of the sample experienced at least one exposure to violence at baseline.

Neighborhood conditions. Neighborhood conditions were measured with a 21-item scale [35] using a four-point scale ranging from “Never” to “Often”. Items asked youth to report on frequency of signs of physical and social disorder in their neighborhood (e.g., “How often do you see graffiti or tags?”, “How often do you see people using needles or syringes to take drugs?”). Responses were averaged from the baseline interview (*M* = 2.1, *SD* = 0.7, α = 0.93).

School orientation. Participants’ orientation toward school was assessed with a 7-item scale [36] that evaluated youths’ commitment to academics (e.g., “Schoolwork is very important to me”). Responses were averaged from a five-point Likert Scale ranging from 1 (“Strongly Disagree”) to 5 (“Strong Agree”) from the baseline interview (*M* = 3.9, *SD* = 0.6, α = 0.80).

Parental monitoring. To address the potential influence of parental supervision, youth responded to a 4-item scale [37] that assesses how much they believe their parents tried to monitor their behavior (e.g., “How much does your parent try to know about how you spend your free time?”). Youth responded using a four-point scale ranging from 1 (“Doesn’t try at all”) to 4 (“Tries extremely hard”) and responses were averaged at baseline (*M* = 3.2, *SD* = 0.7, α = 0.62).

Peer delinquency. Peer delinquency was measured with a 20-item scale [38] that assessed how many of the youths’ friends engaged in delinquent behavior (e.g., “How many of your friends have gotten into a physical fight?”) or encouraged delinquent behavior (e.g., “How many of your friends have suggested you should steal something?”). Responses at baseline were averaged using a five-point scale ranging from 1 (“None of them”) to 5 (“All of them”) at baseline (*M* = 1.6, *SD* = 0.6, α = 0.92).

#### 2.3.4. Risky Behaviors and Condom Use

Offending history. To address the frequently observed link between antisocial behaviors and being a teen father, history of offending behavior was assessed using a 24-item self-report scale (SRO; [39]) that counted the number of types of crimes youth had ever engaged in. At baseline, participants responded to each item (e.g., “Have you ever destroyed or damaged property that did not belong to you?”, “Have you ever driven drunk or high?”) with a “yes” (1) or “no” (0). Responses were counted and summed from the baseline interview (*M* = 3.4, *SD* = 3.1).

Substance use frequency. Youth also self-reported their highest frequency use of different substances [40] including alcohol, cigarettes, marijuana, and 10 other illicit drugs (e.g., cocaine, heroin), for a total of 12 items. Youth responded to items (e.g., “What is the most that you ever used marijuana?”) using a nine-point scale ranging from 1 (“Not at all”) to 9 (“Every day”). Responses were averaged from the baseline interview (*M* = 4.6, *SD* = 3.3). More than 20% of the sample reported engaging in some type of substance use every day, with 40% reporting substance use at least once a week.

Condom use. Among youth who reported having ever had vaginal sex at baseline (49.9% of the sample), a follow-up question about condom use asked youth, “Thinking about the past six months, when you have had vaginal sex, how often did you use a condom?” Youth responded on a four-point scale ranging from “Never” (1) to “Always” (4) (*M* = 3.2, *SD* = 1.0). Approximately 52.6% of youth who were sexually active said they “always” used condoms, 21.7% of youth said they used condoms “most of the time,” 17.8% indicated they used condoms “sometimes,” and approximately 7.9% said they “never” used condoms. 

Demographic covariates. Youth self-reported their race at the baseline interview, and it was coded as a dummy variable with four categories: Hispanic, Black, White, and Other. Age at first arrest was calculated based on youths’ dates of birth. Youth self-reported their parents’ highest level of education, which was used as a proxy for socioeconomic status (SES). Note that because a chi-square test indicated that there were no differences in rates of teen fatherhood across the three different study sites, site was not included as a covariate.

### 2.4. Analytic Plan

We first identified teen fathers in the sample. As noted above, a youth could qualify as a teen father during any point of the study, as long as he was 19 years old or younger when he first reported “yes” to having a child. Then, using factors from the baseline interview (i.e., factors at first arrest), a series of binary logistic regression models were used to predict the odds that a youth would ever become a teen father.

Similarly to the comprehensive models used by Khurana & Gavazzi, the predictors were organized into three different domains: individual factors, social and contextual factors, and engagement in risky behaviors [16]. If a factor from one of these domains significantly predicted teen fatherhood, it was put into a “significant factors” model which utilized the statistically significant factors from each domain, to help identify which variable(s) most strongly contributed to the prediction of teen fatherhood.

Finally, because use of contraceptives is consistently linked to teen pregnancy rates, condom use was added as a predictor to the significant factors model to determine whether it could account for the predictive utility of the significant risk factors. In other words, condom use was added to help determine if there are factors stronger than contraceptive use that are uniquely linked to teen fatherhood in justice-involved boys. All models controlled for demographic covariates. Analyses were conducted in Stata Version 16.

### 2.5. Missing Data

Sample retention in the present study was high, averaging 88% across seven years of data collection (10 waves). Nearly two-thirds of the sample (63%) had no missing data on the fatherhood variable (“Do you have any children?”) at any wave. However, because the timing of fatherhood (before age 20 vs. After age 20) was central to the analysis, virtually all participants who had ever answered the fatherhood question could be included in the model; only one participant was missing data at all timepoints. Other than the variable for condom use, which was only administered to participants who were sexually active, there was very little missing data on the predictor variables; this is largely because all predictor variables were taken from the baseline interview of the study. Approximately 86% of participants were not missing data on any of the predictor variables, 13% were missing data on one predictor, 13 participants were missing data on two predictors, and two participants were missing data on 3 predictors (the average number of missing predictors across participants was 0.16—less than 1.3% were missing data on more than one predictor). The predictor with the largest amount of missing data was parental monitoring; approximately 5.2% of the sample had missing data on this variable. Regardless, there were no significant associations between having missing data on any predictor variables and the teen fatherhood variable. It is thus unlikely that missing data had a meaningful impact on the results.

## 3. Results

Approximately 30% of the sample (*n* = 368) reported being a father at any point during the study, with half of all fathers (49.7%) reporting becoming a parent before age 20 (*n* = 183; 15% of the total sample). Less than 1% of the sample (*n* = 12) reported being a father at the baseline interview; these participants were excluded from analyses since the temporal ordering of predictive factors and fatherhood could not be ascertained. The final sample consisted of 1204 total youth, with 171 teen fathers. The teen fathers were compared to the rest of the sample.

Descriptive information about the teen fathers relative to the full Crossroads sample is presented in Table 1. These youth ranged in age from 15 to 19 (M = 17.7) when they became fathers, and approximately 47% reported being Hispanic/Latino, 44% Black/African American, 7% White, and 4% of another race. Compared to the full sample, Black/African American youth were slightly more likely to become teen fathers (*χ*^2^ [1, *N* = 1204] = 3.87, *p* = 0.049) while White youth were less likely to become teen fathers (*χ*^2^ [1, *N* = 1204] = 9.86, *p* = 0.002). There were no differences between groups in age at first arrest. Socioeconomic status of teen fathers was largely similar to the full sample, with two-thirds of teen fathers reporting having at least one parent who had completed high school or greater, and one-third reporting parents who had not completed a high school diploma.

Results from logistic regression models predicting the likelihood of becoming a teen father can be found in Table 2. Significant findings are presented below as odds ratios (OR); an OR that is less than 1 corresponds to a decreased likelihood of an event, while an OR above 1 corresponds to an increased likelihood of an event occurring.

### 3.1. Individual Factors

Impulse control, sensation seeking, future orientation, and self-esteem were entered simultaneously into the first model. None of the individual factors were associated with teen fatherhood above and beyond demographic covariates.

### 3.2. Social and Contextual Factors

Exposure to violence, neighborhood conditions, school orientation, parental monitoring, and peer delinquency were entered simultaneously into a second model. Poorer neighborhood conditions were marginally associated with increased odds that youth would become a teen father (OR = 1.35, *p* = 0.049), as well as having a higher number of delinquent peers (OR = 1.53, *p* = 0.02). Exposure to violence, school orientation, and parental monitoring were not significantly related to the odds of becoming a teen father.

### 3.3. Risky Behaviors

Offending history and substance use frequency were entered simultaneously into a third model. Both greater offending (OR = 1.07, *p* = 0.016) and greater frequency of substance use (OR = 1.10, *p* = 0.005) were associated with increased odds of becoming a teen father.

### 3.4. Significant Factors Model

Using the statistically significant factors from the three domain models, neighborhood conditions, peer delinquency, offending, and substance use were entered simultaneously in a fourth and final model along with demographic covariates. In this model, results indicated that only substance use was significantly associated with becoming a teen father, such that those who used substances more frequently had increased odds of becoming a teen father (OR = 1.09, *p* = 0.018).

This model was then estimated again including condom use as a covariate. Greater frequency of condom use was linked to decreased odds of becoming a teen father (OR = 0.74, *p* = 0.006). With the inclusion of condom use, all other predictors failed to reach statistical significance.

## 4. Discussion

In 2020 alone, more than 420,000 teenage boys were arrested [41]. While a first arrest is an undoubtedly negative event in these boys’ lives, it may also afford policymakers an opportunity to help prevent teen fatherhood in a population that is high risk. Though prior work has identified commonalities among justice-involved teen fathers, little research has explored what factors might prospectively indicate a heightened (or reduced) likelihood of becoming a father during adolescence. To address this gap in the literature, our study examined whether factors at the time of a youth’s first arrest predicted the likelihood of fathering a child during adolescence.

It is notable that teen fathers were demographically similar to non-teen fathers in the sample. Consistent with prior research [21], Black youth comprised a slightly higher proportion of teen fathers, and they were also more likely to become fathers in general (regardless of age at parenthood). White youth were less likely to become teen fathers, and they were also less likely to become a father at all. While there was no greater statistical likelihood that Hispanic youth would become a teen father, identifying as Hispanic (as opposed to White) was a significant predictor in the majority of the models. This suggests that Hispanic youth may experience more risk factors and be at higher risk of becoming a teen dad than White youth. Meanwhile, teen fathers were no different from their peers in age at first arrest or socioeconomic status. This suggests that demographically, all justice-involved boys are largely at equally increased risk of becoming a teenage parent—the 15% rate of teen fathers in our sample is more than double of the 6.2% of fathers that are between the ages of 15 to 24 in the general U.S. population [42].

Interestingly, none of the individual-level factors significantly predicted teen fatherhood. In particular, given that adolescence is characterized by heightened sensation seeking and impulsivity [17], it is surprising that our findings were inconsistent with previous studies emphasizing the role of impulsive decision-making and sensation seeking in unsafe sexual behaviors [20]. However, because factors such as impulse control are consistently related to riskier sex in other studies, individual factors may remain nonetheless important for establishing safe sexual behaviors. Considering social and contextual factors, having poorer neighborhood conditions and friends who engaged in delinquency increased the likelihood that youth would become teen fathers. Unlike prior work with justice-involved boys [24], exposure to violence did not significantly predict teen fatherhood, indicating violence exposure may not increase risk when other social and contextual factors are taken into account. Similarly, parental monitoring and school orientation do not appear to be linked to teen fatherhood above and beyond other factors. Finally, consistent with previous literature [16,26], this study identified that the risky behaviors of offending and substance use were both related to teen fatherhood.

When taking all significant factors into account, teen fatherhood appears to be especially driven by the frequency of substance use. Thus, while the other factors may help identify which youth are most at risk of becoming an adolescent parent, their effects may be due to their influence on the youth’s substance use. Further, when condom use was included, the association between substance use and teen fatherhood disappeared. In other words, substance use may increase the risk of teen fatherhood because it leads to riskier sexual behaviors, which is in turn linked to teen parenthood. Thus, increasing condom use may have the largest impact on preventing teenage pregnancies among justice-involved boys, with more frequent use of condoms reducing the odds of teen fatherhood by approximately 26%. Nonetheless, policy discussions surrounding increasing condom use should consider the role of substance use on safe sex practices.

Interventions that not only provide information about sexually transmitted infections and pregnancy prevention, but also address social attitudes and offer behavioral skills training (e.g., negotiating condom use with a partner) are frequently found to be effective in increasing condom use [43]. Sexually active high school students are also more likely to use condoms when they are distributed at school, and despite potential concerns, this condom distribution is not associated with initiation of sexual activity [44]. In other words, distributing condoms means that adolescents who are already having sex will have safer sex. Having condoms readily available can also reduce the chance an adolescent will be unprepared for a sexual encounter while drinking alcohol or using other substances. Further, multi-domain approaches that incorporate the family, school, and community, such as the Seattle Social Development Project (SSDP), have had significant effects on reducing both substance use and risky sexual behaviors [45]. As such, supporting condom use interventions that address multiple ecological factors may be most effective in reducing both substance use and teen fatherhood.

These programs also need to consider the reasons that teens may not use condoms. Teens are usually aware of the risks of not wearing condoms (pregnancy, sexually transmitted infections), but they report not using them because of the potential for reduced sexual pleasure [46]. This is in line with research showing that adolescents are more sensitive to rewards than punishments [47] and sensitivity to potential pleasure could be enhanced by substance use [48]. Advertising tactics that associate condom use with positive lifestyle messages have been found to increase condom use, as well as lessen embarrassment about purchasing condoms compared to advertisements that relay fear-inducing messages [49]. Similarly, curriculum which emphasizes learning about the pleasurable components of sex, rather than only reproduction, has been found to reduce teen pregnancies [50]. Strategies that implement positive messaging may be particularly effective for justice-involved youth, who tend to show greater preference for immediate rewards when making decisions relative to community youth [51]. Future policy initiatives should thus aim to not only increase access to condoms, but also build educational programs which highlight pleasurable aspects of safe sex.

## 5. Limitations and Future Directions

These findings should be considered in light of the present study’s limitations. It is important to acknowledge that the multi-contextual factors captured at baseline may change over time. However, the goal of this current study is to identify baseline risk factors that can inform practitioners and policymakers when considering interventions at a teen’s initial contact with the justice system. Additionally, the study data at baseline only included subjects with low-level crimes at their first arrest. There is evidence showing young men who rarely engage in offending behaviors are less likely to partake in risky sexual behaviors and substance use compared to youth with chronic, higher-level offenses [52]. Thus, this study could also further benefit from understanding how these multi-contextual factors relate to teen fatherhood among young men with violent or high-level offenses.

Moreover, measures relied on self-reports and reflected youth’s perception of each factor. Incorporating objective indicators (e.g., community disadvantage, health or administrative records) could offer a clearer and more accurate understanding of underlying risks. Although, it is important to note that relying solely on official records may overlook youth’s unique perspectives of contextual factors, such as neighborhood connectedness and collective efficacy [53], which alternatively may serve as protective factors. As such, future research may benefit most from multi-method approaches that incorporate both self-report and objective measures in investigating these associations. Sipsma and colleagues’ study also highlight that sons of teen fathers were 1.8 times more likely to become teen fathers themselves [4]. Although this study did not collect data on whether participants’ parents were teen parents, considering this additional risk factor in the future could further clarify which individuals are at greater risk of becoming teen fathers.

Further, this study captures teen fatherhood, not teen pregnancy, and risk factors for teen pregnancies that are brought to term may be different than teen pregnancies that do not end in live birth. Additionally, we do not have details on whether or not these were planned pregnancies. Capturing these details in future studies may provide a more nuanced understanding of youth’s reproductive behaviors and associated outcomes. Nonetheless, data suggests that the majority of teen pregnancies are unplanned [2], and while a small minority of adolescent girls report wanting to become pregnant (e.g., [54]), there is very little if any research on rates of planned adolescent pregnancies among males. Finally, because the rate of teen fatherhood is relatively low in the sample, this may limit the statistical power of the model. Nonetheless, the number of predictors (no more than 10 per model) exceeds the general “events per variable” guideline for logistic regression, limiting bias in the results [55].

## 6. Conclusions

This study suggests that increasing condom use may reduce adolescent parenthood among justice-involved boys, with strategies that are largely similar to those employed with community youth. Interventions which also address substance use and being prepared for sexual encounters in situations where substances are available, may also help target the high observed rates of teen fatherhood. By focusing resources on boys who are involved in the justice system from their first point of contact, there is potential to promote positive psychosocial and health outcomes in an at-risk population that demonstrates elevated rates of teen parenthood.

## Figures and Tables

**Table 1:** Demographics of the entire Crossroads sample (*N* = 1216) and analytic sample of teen fathers in the Crossroads sample (*n* = 171).

	Full Sample		Teen Fathers	
	%	*N*	%	*n*
Race				
Hispanic	45.8	557	46.8	80
Black	36.9	449	43.9	75
White	14.8	180	7.0	12
Other	2.5	30	2.3	4
Socioeconomic Status				
At least a high school diploma	67.9	826	66.7	106
Less than a high school diploma	28.0	340	33.3	53
Unsure/unknown	4.1	50	-	-
	*M*	*SD*	*M*	*SD*
Age at Baseline	15.3	1.29	15.1	1.22

**Table 2:** Results from logistic regression models predicting the odds of becoming a teen father.

Individual Predictors	OR	SE	95% CI
Impulse Control	0.90	0.12	[0.70, 1.19]
Sensation Seeking	0.99	0.06	[0.88, 1.12]
Future Orientation	0.91	0.16	[0.65, 1.27]
Self Esteem	1.00	0.02	[0.96, 1.04]
Age	0.93	0.06	[0.82, 1.06]
Socioeconomic Status	0.82	0.16	[0.56, 1.21]
Black v. White	2.77 **	0.92	[1.44, 5.30]
Hispanic v. White	2.11 *	0.71	[1.10, 4.07]
Other v. White	1.66	1.13	[0.44, 6.31]
Other v. White	1.84	1.30	[0.46, 7.31]
**Social/Contextual Predictors**	OR	SE	95% CI
Exposure to Violence	1.04	0.04	[0.96, 1.12]
Neighborhood Conditions	1.35 *	0.20	[1.00, 1.81]
School Orientation	0.88	0.14	[0.64, 1.20]
Parental Monitoring	1.26	0.19	[0.93, 1.70]
Peer Delinquency	1.53 *	0.27	[1.09, 2.15]
Age	0.88	0.07	[0.76, 1.02]
Socioeconomic Status	0.75	0.16	[0.49, 1.14]
Black v. White	2.38 *	0.86	[1.17, 4.85]
Hispanic v. White	1.80	0.65	[0.89, 3.63]
Other v. White	1.84	1.30	[0.46, 7.31]
**Risky Behaviors**	OR	SE	95% CI
Offending History	1.07 *	0.03	[1.01, 1.13]
Substance Use Frequency	1.10 **	0.04	[1.03, 1.17]
Age	0.81 **	0.06	[0.70, 0.94]
Socioeconomic Status	0.80	0.16	[0.54, 1.18]
Black v. White	3.26 ***	1.09	[1.69, 6.26]
Hispanic v. White	2.30 *	0.77	[1.19, 4.44]
Other v. White	1.85	1.27	[0.48, 7.10]
**Significant Factors Model**	OR	SE	95% CI
Neighborhood Conditions	1.27	0.18	[0.97, 1.66]
Peer Delinquency	1.06	0.20	[0.73, 1.55]
Offending History	1.05	0.04	[0.98, 1.13]
Substance Use Frequency	1.09 *	0.04	[1.01, 1.16]
Age	0.83 *	0.06	[0.71, 0.96]
Socioeconomic Status	0.81	0.16	[0.55, 1.20]
Black v. White	2.89 **	0.99	[1.48, 5.66]
Hispanic v. White	2.17 *	0.74	[1.12, 4.23]
Other v. White	1.78	1.22	[0.46, 6.84]
**Significant Factors Model Including Condom Use**	OR	SE	95% CI
Neighborhood Conditions	1.32	0.22	[0.94, 1.84]
Peer Delinquency	0.76	0.19	[0.47, 1.24]
Offending History	1.06	0.04	[0.98, 1.15]
Substance Use Frequency	1.07	0.05	[0.98, 1.17]
Condom Use	0.74 **	0.08	[0.59, 0.92]
Age	0.74 **	0.07	[0.61, 0.90]
Socioeconomic Status	0.51 *	0.13	[0.31, 0.86]
Black v. White	2.63 *	1.10	[1.16, 5.96]
Hispanic v. White	1.44	0.60	[0.64, 3.25]
Other v. White	0.93	0.78	[0.18, 4.83]

OR = Odds ratio. SE = Standard error. CI = Confidence interval. * *p* < 0.05, ** *p* < 0.01, *** *p* < 0.001. Bolding is used here to highlight the different domains/models.

## Data Availability

Data will be archived with the National Institute of Justice upon completion of the larger study (see Cauffman et al. [29]). Data and materials requests can be made to the senior author.
